# Supplementary material for: Resolving the full spectrum of human genome variation using Linked-Reads
Source: Genome Res. 2019 Apr;29(4):635–45. doi: 10.1101/gr.234443.118 (PMC6442396; doi:10.1101/gr.234443.118)
Supplement: Supplemental Material [file supp_gr.234443.118_Supplemental_longranger-2.2.zip › longranger-2.2.tar.bz2!/lr2.2-public/lib/stan/cmdstan/stan/doc/api/html/annotated.html]

Stan: Class List


|  |  |
| --- | --- |
|  | Stan  2.5.0  probability, sampling & optimization |


- Main Page
- Related Pages
- Namespaces
- Classes
- Files

- Class List
- Class Index
- Class Hierarchy
- Class Members


All Classes Namespaces Files Functions Variables Typedefs Enumerations Enumerator Friends Macros Pages

Class List

Here are the classes, structs, unions and interfaces with brief descriptions:

[detail level 1234]

|  |  |
| --- | --- |
| boost | Reimplementing boost functionality |
| math | Reimplmeneting boost functionality for `stan::agrad::var` and and bugs in classification of integer types |
| Eigen | (Expert) Numerical traits for algorithmic differentiation variables |
| internal | (Expert) Product traits for algorithmic differentiation variables |
| significant\_decimals\_default\_impl< stan::agrad::var, false > | Implemented this for printing to stream |
| scalar\_product\_traits< stan::agrad::var, double > | Scalar product traits override for Eigen for automatic gradient variables |
| scalar\_product\_traits< double, stan::agrad::var > | Scalar product traits override for Eigen for automatic gradient variables |
| general\_matrix\_vector\_product< Index, stan::agrad::var, ColMajor, ConjugateLhs, stan::agrad::var, ConjugateRhs > | Override matrix-vector and matrix-matrix products to use more efficient implementation |
| general\_matrix\_vector\_product< Index, stan::agrad::var, RowMajor, ConjugateLhs, stan::agrad::var, ConjugateRhs > |  |
| general\_matrix\_matrix\_product< Index, stan::agrad::var, LhsStorageOrder, ConjugateLhs, stan::agrad::var, RhsStorageOrder, ConjugateRhs, ColMajor > |  |
| NumTraits< stan::agrad::var > | Numerical traits template override for Eigen for automatic gradient variables |
| stan | Probability, optimization and sampling library |
| agrad | Function gradients via reverse-mode automatic differentiation |
| fvar |  |
| partials\_vari |  |
| OperandsAndPartials | A variable implementation that stores operands and derivatives with respect to the variable |
| chainable | Abstract base class for variable implementations that handles memory management and applying the chain rule |
| op\_ddv\_vari |  |
| op\_dv\_vari |  |
| op\_dvd\_vari |  |
| op\_dvv\_vari |  |
| op\_matrix\_vari |  |
| precomp\_v\_vari |  |
| precomputed\_gradients\_vari | This is a var implementation class that takes precomputed gradient values |
| op\_v\_vari |  |
| op\_vd\_vari |  |
| op\_vdd\_vari |  |
| op\_vdv\_vari |  |
| op\_vector\_vari |  |
| op\_vv\_vari |  |
| op\_vvd\_vari |  |
| op\_vvv\_vari |  |
| LDLT\_alloc | This object stores the actual (double typed) LDLT factorization of an Eigen::Matrix<var> along with pointers to its vari's which allow the \*\_ldlt functions to save memory |
| stored\_gradient\_vari | A var implementation that stores the daughter variable implementation pointers and the partial derivative with respect to the result explicitly in arrays constructed on the auto-diff memory stack |
| var | Independent (input) and dependent (output) variables for gradients |
| chainable\_alloc | A chainable\_alloc is an object which is constructed and destructed normally but the memory lifespan is managed along with the arena allocator for the gradient calculation |
| vari | The variable implementation base class |
| common |  |
| recorder |  |
| csv | Writes out a vector as string |
| filtered\_values |  |
| messages |  |
| no\_op | Does nothing |
| sum\_values |  |
| values |  |
| NoOpFunctor |  |
| var\_context\_factory |  |
| dump\_factory |  |
| gm | Directed graphical model compiler |
| arg\_adapt |  |
| arg\_adapt\_delta |  |
| arg\_adapt\_engaged |  |
| arg\_adapt\_gamma |  |
| arg\_adapt\_init\_buffer |  |
| arg\_adapt\_kappa |  |
| arg\_adapt\_t0 |  |
| arg\_adapt\_term\_buffer |  |
| arg\_adapt\_window |  |
| arg\_bfgs |  |
| arg\_data |  |
| arg\_data\_file |  |
| arg\_dense\_e |  |
| arg\_diag\_e |  |
| arg\_diagnose |  |
| arg\_diagnostic\_file |  |
| arg\_engine |  |
| arg\_fail |  |
| arg\_fixed\_param |  |
| arg\_history\_size |  |
| arg\_hmc |  |
| arg\_id |  |
| arg\_init |  |
| arg\_init\_alpha |  |
| arg\_int\_time |  |
| arg\_iter |  |
| arg\_lbfgs |  |
| arg\_max\_depth |  |
| arg\_method |  |
| arg\_metric |  |
| arg\_newton |  |
| arg\_num\_samples |  |
| arg\_num\_warmup |  |
| arg\_nuts |  |
| arg\_optimize |  |
| arg\_optimize\_algo |  |
| arg\_output |  |
| arg\_output\_file |  |
| arg\_random |  |
| arg\_refresh |  |
| arg\_rwm |  |
| arg\_sample |  |
| arg\_sample\_algo |  |
| arg\_save\_iterations |  |
| arg\_save\_warmup |  |
| arg\_seed |  |
| arg\_static |  |
| arg\_stepsize |  |
| arg\_stepsize\_jitter |  |
| arg\_test |  |
| arg\_test\_grad\_eps |  |
| arg\_test\_grad\_err |  |
| arg\_test\_gradient |  |
| arg\_thin |  |
| arg\_tolerance |  |
| arg\_unit\_e |  |
| argument |  |
| argument\_parser |  |
| argument\_probe |  |
| categorical\_argument |  |
| list\_argument |  |
| type\_name |  |
| type\_name< int > |  |
| type\_name< unsigned int > |  |
| type\_name< double > |  |
| type\_name< bool > |  |
| type\_name< std::string > |  |
| singleton\_argument |  |
| unvalued\_argument |  |
| valued\_argument |  |
| nil | Placeholder struct for boost::variant default ctors |
| expr\_type |  |
| function\_signatures |  |
| statements |  |
| distribution |  |
| expression\_type\_vis |  |
| expression |  |
| printable |  |
| is\_nil\_op |  |
| variable\_dims |  |
| int\_literal |  |
| double\_literal |  |
| array\_literal |  |
| variable |  |
| integrate\_ode |  |
| fun |  |
| index\_op |  |
| binary\_op |  |
| unary\_op |  |
| range |  |
| base\_var\_decl |  |
| variable\_map |  |
| int\_var\_decl |  |
| double\_var\_decl |  |
| unit\_vector\_var\_decl |  |
| simplex\_var\_decl |  |
| ordered\_var\_decl |  |
| positive\_ordered\_var\_decl |  |
| vector\_var\_decl |  |
| row\_vector\_var\_decl |  |
| matrix\_var\_decl |  |
| cholesky\_factor\_var\_decl |  |
| cholesky\_corr\_var\_decl |  |
| cov\_matrix\_var\_decl |  |
| corr\_matrix\_var\_decl |  |
| name\_vis |  |
| var\_decl |  |
| statement |  |
| is\_no\_op\_statement\_vis |  |
| returns\_type\_vis |  |
| increment\_log\_prob\_statement |  |
| for\_statement |  |
| conditional\_statement |  |
| while\_statement |  |
| print\_statement |  |
| reject\_statement |  |
| return\_statement |  |
| no\_op\_statement |  |
| arg\_decl |  |
| function\_decl\_def |  |
| function\_decl\_defs |  |
| program |  |
| sample |  |
| assignment |  |
| contains\_var |  |
| contains\_nonparam\_var |  |
| error\_codes |  |
| visgen | Generic visitor with output for extension |
| expression\_visgen |  |
| printable\_visgen |  |
| var\_size\_validating\_visgen |  |
| var\_resizing\_visgen |  |
| init\_local\_var\_visgen |  |
| validate\_var\_decl\_visgen |  |
| member\_var\_decl\_visgen |  |
| local\_var\_decl\_visgen |  |
| generate\_local\_var\_init\_nan\_visgen |  |
| generate\_init\_vars\_visgen |  |
| validate\_transformed\_params\_visgen |  |
| statement\_visgen |  |
| dump\_member\_var\_visgen |  |
| set\_param\_ranges\_visgen |  |
| generate\_init\_visgen |  |
| write\_dims\_visgen |  |
| write\_param\_names\_visgen |  |
| write\_csv\_header\_visgen |  |
| constrained\_param\_names\_visgen |  |
| unconstrained\_param\_names\_visgen |  |
| write\_csv\_visgen |  |
| write\_csv\_vars\_visgen |  |
| write\_array\_visgen |  |
| write\_array\_vars\_visgen |  |
| bare\_type\_grammar |  |
| term\_grammar |  |
| expression\_grammar |  |
| expression07\_grammar |  |
| validate\_expr\_type3 |  |
| result |  |
| set\_fun\_type3 |  |
| result |  |
| addition\_expr3 |  |
| result |  |
| subtraction\_expr3 |  |
| result |  |
| set\_fun\_type2 |  |
| result |  |
| binary\_op\_expr |  |
| result |  |
| functions\_grammar |  |
| validate\_non\_void\_arg\_function |  |
| result |  |
| set\_void\_function |  |
| result |  |
| set\_allows\_sampling\_origin |  |
| result |  |
| validate\_declarations |  |
| result |  |
| add\_function\_signature |  |
| result |  |
| validate\_return\_type |  |
| result |  |
| scope\_lp |  |
| result |  |
| unscope\_variables |  |
| result |  |
| add\_fun\_var |  |
| result |  |
| program\_grammar |  |
| statement\_grammar |  |
| statement\_2\_grammar |  |
| add\_conditional\_condition |  |
| result |  |
| add\_conditional\_body |  |
| result |  |
| validate\_return\_allowed |  |
| result |  |
| validate\_void\_return\_allowed |  |
| result |  |
| validate\_assignment |  |
| result |  |
| validate\_sample |  |
| result |  |
| expression\_as\_statement |  |
| result |  |
| unscope\_locals |  |
| result |  |
| add\_while\_condition |  |
| result |  |
| add\_while\_body |  |
| result |  |
| add\_loop\_identifier |  |
| result |  |
| remove\_loop\_identifier |  |
| result |  |
| validate\_int\_expr2 |  |
| result |  |
| validate\_allow\_sample |  |
| result |  |
| validate\_non\_void\_expression |  |
| result |  |
| validate\_integrate\_ode |  |
| result |  |
| set\_fun\_type |  |
| result |  |
| set\_fun\_type\_named |  |
| result |  |
| exponentiation\_expr |  |
| result |  |
| multiplication\_expr |  |
| result |  |
| division\_expr |  |
| result |  |
| modulus\_expr |  |
| result |  |
| left\_division\_expr |  |
| result |  |
| elt\_multiplication\_expr |  |
| result |  |
| elt\_division\_expr |  |
| result |  |
| negate\_expr |  |
| result |  |
| logical\_negate\_expr |  |
| result |  |
| transpose\_expr |  |
| result |  |
| add\_expression\_dimss |  |
| result |  |
| set\_var\_type |  |
| result |  |
| validate\_int\_expr3 |  |
| result |  |
| var\_decls\_grammar |  |
| whitespace\_grammar |  |
| io | Input/output utilities |
| cmd\_line | Parses and stores command-line arguments |
| csv\_writer | Writes Stan variables in comma-separated-value format to an output stream |
| dump\_writer | Writes data into the S-plus dump format |
| dump\_reader | Reads data from S-plus dump format |
| dump | Represents named arrays with dimensions |
| mcmc\_writer | Mcmc\_writer writes out headers and samples |
| reader | A stream-based reader for integer, scalar, vector, matrix and array data types, with Jacobian calculations |
| stan\_csv\_metadata |  |
| stan\_csv\_adaptation |  |
| stan\_csv\_timing |  |
| stan\_csv |  |
| stan\_csv\_reader | Reads from a Stan output csv file |
| var\_context | A `var_reader` reads array variables of integer and floating point type by name and dimension |
| writer | A stream-based writer for integer, scalar, vector, matrix and array data types, which transforms from constrained to a sequence of constrained variables |
| json |  |
| json\_data | A `json_data` is a `var_context` object that represents a set of named values which are typed to either `double` or `int` and can be either scalar value or a non-empty array of values of any dimensionality |
| json\_data\_handler | A `json_data_handler` is an implementation of a `json_handler` that restricts the allowed JSON text a set of Stan variable declarations in JSON format |
| json\_error | Exception type for JSON errors |
| json\_handler | Abstract base class for JSON handlers |
| math | Matrices and templated mathematical functions |
| detail |  |
| bounded |  |
| bounded< T\_y, T\_low, T\_high, T\_result, true > |  |
| policies | Extending boost functionality |
| detail | Reimplementing boost::math::policies::detail::raise\_domain\_error |
| LDLT\_factor< stan::agrad::var, R, C > | A template specialization of src/stan/math/matrix/LDLT\_factor.hpp for stan::agrad::var which can be used with all the \*\_ldlt functions |
| coupled\_ode\_system< F, double, stan::agrad::var > | The coupled ODE system for known initial values and unknown parameters |
| coupled\_ode\_system< F, stan::agrad::var, double > | The coupled ODE system for unknown initial values and known parameters |
| coupled\_ode\_system< F, stan::agrad::var, stan::agrad::var > | The coupled ode system for unknown intial values and unknown parameters |
| promote\_scalar\_struct | General struct to hold static function for promoting underlying scalar types |
| promote\_scalar\_struct< T, T > | Struct to hold static function for promoting underlying scalar types |
| promote\_scalar\_struct< T, std::vector< S > > | Struct to hold static function for promoting underlying scalar types |
| promote\_scalar\_type | Template metaprogram to calculate a type for converting a convertible type |
| promote\_scalar\_type< T, std::vector< S > > | Template metaprogram to calculate a type for a container whose underlying scalar is converted from the second template parameter type to the first |
| accumulator | Class to accumulate values and eventually return their sum |
| array\_builder | Structure for building up arrays in an expression (rather than in statements) using an argumentchaining add() method and a getter method array() to return the result |
| common\_type |  |
| common\_type< std::vector< T1 >, std::vector< T2 > > |  |
| common\_type< Eigen::Matrix< T1, R, C >, Eigen::Matrix< T2, R, C > > |  |
| LDLT\_factor |  |
| LDLT\_factor< T, R, C > | LDLT\_factor is a thin wrapper on Eigen::LDLT to allow for reusing factorizations and efficient autodiff of things like log determinants and solutions to linear systems |
| index\_type< Eigen::Matrix< T, R, C > > | Template metaprogram defining typedef for the type of index for an Eigen matrix, vector, or row vector |
| value\_type< Eigen::Matrix< T, R, C > > | Template metaprogram defining the type of values stored in an Eigen matrix, vector, or row vector |
| promote\_scalar\_struct< T, Eigen::Matrix< S,-1,-1 > > | Struct to hold static function for promoting underlying scalar types |
| promote\_scalar\_struct< T, Eigen::Matrix< S, 1,-1 > > | Struct to hold static function for promoting underlying scalar types |
| promote\_scalar\_struct< T, Eigen::Matrix< S,-1, 1 > > | Struct to hold static function for promoting underlying scalar types |
| promote\_scalar\_type< T, Eigen::Matrix< S, Eigen::Dynamic, Eigen::Dynamic > > | Template metaprogram to calculate a type for a matrix whose underlying scalar is converted from the second template parameter type to the first |
| promote\_scalar\_type< T, Eigen::Matrix< S, Eigen::Dynamic, 1 > > | Template metaprogram to calculate a type for a vector whose underlying scalar is converted from the second template parameter type to the first |
| promote\_scalar\_type< T, Eigen::Matrix< S, 1, Eigen::Dynamic > > | Template metaprogram to calculate a type for a row vector whose underlying scalar is converted from the second template parameter type to the first |
| promoter |  |
| promoter< T, T > |  |
| promoter< std::vector< F >, std::vector< T > > |  |
| promoter< std::vector< T >, std::vector< T > > |  |
| promoter< Eigen::Matrix< F, R, C >, Eigen::Matrix< T, R, C > > |  |
| promoter< Eigen::Matrix< T, R, C >, Eigen::Matrix< T, R, C > > |  |
| index\_type | Primary template class for the metaprogram to compute the index type of a container |
| index\_type< const T > | Template class for metaprogram to compute the type of indexes used in a constant container type |
| index\_type< std::vector< T > > | Template metaprogram class to compute the type of index for a standard vector |
| value\_type | Primary template class for metaprogram to compute the type of values stored in a container |
| value\_type< const T > | Template class for metaprogram to compute the type of values stored in a constant container |
| value\_type< std::vector< T > > | Template metaprogram class to compute the type of values stored in a standard vector |
| coupled\_ode\_observer | Observer for the coupled states |
| coupled\_ode\_system | Base template class for a coupled ordinary differential equation system, which adds sensitivities to the base system |
| coupled\_ode\_system< F, double, double > | The coupled ode system for known initial values and known parameters |
| store\_type |  |
| store\_type< double > |  |
| store\_type< int > |  |
| pass\_type |  |
| pass\_type< double > |  |
| pass\_type< int > |  |
| seq\_view |  |
| seq\_view< T, Eigen::Matrix< S, Eigen::Dynamic, 1 > > |  |
| seq\_view< T, Eigen::Matrix< S, 1, Eigen::Dynamic > > |  |
| seq\_view< T, Eigen::Matrix< S, Eigen::Dynamic, Eigen::Dynamic > > |  |
| seq\_view< T, std::vector< S > > |  |
| seq\_view< T, std::vector< T > > |  |
| seq\_view< T, std::vector< std::vector< T > > > |  |
| seq\_view< double, std::vector< int > > |  |
| mcmc | Markov chain Monte Carlo samplers |
| base\_adaptation |  |
| base\_adapter |  |
| base\_mcmc |  |
| chains | An `mcmc::chains` object stores parameter names and dimensionalities along with samples from multiple chains |
| covar\_adaptation |  |
| fixed\_param\_sampler |  |
| base\_hmc |  |
| base\_hamiltonian |  |
| dense\_e\_metric |  |
| dense\_e\_point |  |
| diag\_e\_metric |  |
| diag\_e\_point |  |
| ps\_point |  |
| unit\_e\_metric |  |
| unit\_e\_point |  |
| base\_integrator |  |
| base\_leapfrog |  |
| expl\_leapfrog |  |
| adapt\_dense\_e\_nuts |  |
| adapt\_diag\_e\_nuts |  |
| adapt\_unit\_e\_nuts |  |
| nuts\_util |  |
| base\_nuts |  |
| dense\_e\_nuts |  |
| diag\_e\_nuts |  |
| unit\_e\_nuts |  |
| adapt\_dense\_e\_static\_hmc |  |
| adapt\_diag\_e\_static\_hmc |  |
| adapt\_unit\_e\_static\_hmc |  |
| base\_static\_hmc |  |
| dense\_e\_static\_hmc |  |
| diag\_e\_static\_hmc |  |
| unit\_e\_static\_hmc |  |
| sample |  |
| stepsize\_adaptation |  |
| stepsize\_adapter |  |
| stepsize\_covar\_adapter |  |
| stepsize\_var\_adapter |  |
| var\_adaptation |  |
| windowed\_adaptation |  |
| memory | (Expert) Allocate, free, and manage underlying system memory |
| stack\_alloc | An instance of this class provides a memory pool through which blocks of raw memory may be allocated and then collected simultaneously |
| model | For compiling models |
| prob\_grad | The `prob_grad` class represents the basic parameter holders for a model |
| model\_functional |  |
| optimization |  |
| ConvergenceOptions |  |
| LSOptions |  |
| BFGSMinimizer |  |
| ModelAdaptor |  |
| BFGSLineSearch |  |
| BFGSUpdate\_HInv |  |
| LBFGSUpdate | Implement a limited memory version of the BFGS update |
| prob | Templated probability distributions |
| include\_summand | Template metaprogram to calculate whether a summand needs to be included in a proportional (log) probability calculation |
| welford\_covar\_estimator |  |
| welford\_var\_estimator |  |
| error\_index |  |
| is\_constant | Metaprogramming struct to detect whether a given type is constant in the mathematical sense (not the C++ `const` sense) |
| is\_constant\_struct | Metaprogram to determine if a type has a base scalar type that can be assigned to type double |
| is\_constant\_struct< std::vector< T > > |  |
| is\_constant\_struct< Eigen::Matrix< T, R, C > > |  |
| is\_constant\_struct< Eigen::Block< T > > |  |
| is\_vector |  |
| is\_vector< const T > |  |
| is\_vector< std::vector< T > > |  |
| is\_vector< Eigen::Matrix< T, Eigen::Dynamic, 1 > > |  |
| is\_vector< Eigen::Matrix< T, 1, Eigen::Dynamic > > |  |
| is\_vector< Eigen::Block< T > > |  |
| scalar\_type | Metaprogram structure to determine the base scalar type of a template argument |
| size\_of\_helper |  |
| size\_of\_helper< T, true > |  |
| scalar\_type< Eigen::Matrix< T, Eigen::Dynamic, Eigen::Dynamic > > |  |
| scalar\_type< T \* > |  |
| is\_vector\_like |  |
| is\_vector\_like< T \* > |  |
| is\_vector\_like< const T > |  |
| is\_vector\_like< Eigen::Matrix< T, Eigen::Dynamic, Eigen::Dynamic > > |  |
| VectorView | VectorView is a template metaprogram that takes its argument and allows it to be used like a vector |
| VectorView< const T, is\_array, throw\_if\_accessed > | VectorView that has const correctness |
| VectorView< const double, false, false > |  |
| DoubleVectorView | DoubleVectorView allocates double values to be used as intermediate values |
| DoubleVectorView< true, false > |  |
| DoubleVectorView< true, true > |  |
| return\_type | Metaprogram to calculate the base scalar return type resulting from promoting all the scalar types of the template parameters |
| is\_fvar |  |
| is\_fvar< stan::agrad::fvar< T > > |  |
| is\_var |  |
| is\_var< stan::agrad::var > |  |
| contains\_fvar | Metaprogram to calculate the base scalar return type resulting from promoting all the scalar types of the template parameters |
| is\_var\_or\_arithmetic |  |
| scalar\_type\_pre | Metaprogram structure to determine the type of first container of the base scalar type of a template argument |
| VectorViewMvt |  |
| VectorViewMvt< const T, is\_array, throw\_if\_accessed > | VectorViewMvt that has const correctness |
| std | Template specification of functions in std for Stan |
| numeric\_limits< stan::agrad::fvar< T > > |  |
| numeric\_limits< stan::agrad::var > | Specialization of numeric limits for var objects |
| add\_var |  |
| result |  |
| copy\_square\_cholesky\_dimension\_if\_necessary |  |
| result |  |
| data\_only\_expression |  |
| empty\_range |  |
| result |  |
| set\_double\_range\_lower |  |
| result |  |
| set\_double\_range\_upper |  |
| result |  |
| set\_int\_range\_lower |  |
| result |  |
| set\_int\_range\_upper |  |
| result |  |
| validate\_decl\_constraints |  |
| result |  |
| validate\_double\_expr |  |
| result |  |
| validate\_identifier |  |
| result |  |
| validate\_int\_data\_expr |  |
| result |  |
| validate\_int\_expr |  |
| result |  |


---

[ Stan Home Page ]

*© 2011–2014,
Stan Development Team.*
